# Bilateral Pseudoaneurysms Following Percutaneous Achilles Tenotomy for Congenital Talipes Equinovarus

**DOI:** 10.7759/cureus.31692

**Published:** 2022-11-20

**Authors:** Rajiv P Doshi, Sandeep Gokhale, Sieu Ming Ng, Clare Carpenter

**Affiliations:** 1 Department of Trauma and Orthopaedics, University Hospital of Wales, Cardiff, GBR; 2 Department of General Surgery, University Hospital of Wales, Cardiff, GBR

**Keywords:** pseudoaneurysm, achilles tendon tenotomy, congenital talipes equinovarus, ponseti, idiopathic clubfoot

## Abstract

Pseudoaneurysm following percutaneous tendoachilles (TA) tenotomy is a rare complication found in children with congenital talipes equinovarus (CTEV). It is postulated that associated aberrant vascular anatomy in combination with CTEV may be the underlying aetiology. In this case report, we describe a case of a toddler who developed bilateral pseudoaneurysms following percutaneous tendoachilles tenotomy and explore the management and outcome in relation to this. Based on this case report and a review of the literature, the consistent clinical findings of swelling and/or discolouration due to pseudoaneurysm occur at three weeks post-tenotomy, and should raise suspicion for the diagnosis. Furthermore to the best of our knowledge, this is the first case report of bilateral pseudoaneurysms in the same setting, and we propose the possibility of an aberrant vessel arising from the peroneal artery that may be prone to injury.

## Introduction

Congenital talipes equinovarus (CTEV), or clubfoot, is one of the most common congenital foot abnormalities. However, its aetiology remains poorly understood. CTEV deformity consists of four main components: the equinus, hindfoot varus, forefoot adductus and cavus. The aim of treatment is to provide a long-term correction to these deformities allowing the patient to achieve a functional, supple, and pain-free foot [1,2]. 

The Ponseti method was first developed by Dr. Ignacio V. Ponseti in 1950 and is now the first-line treatment of choice for CTEV. This method uses manipulation and application of a well-moulded long plaster cast to correct cavus, adduction and varus deformities [3]. In the majority of cases, percutaneous tendoachilles (TA) tenotomy is then required to correct the remaining equinus deformity. It has been shown to reduce the need for extensive surgical intervention compared with other treatment methods [4]. However as with all procedures, it is not without its risks and complications. We present a case of bilateral pseudoaneuryms following Ponseti percutaneous Achilles tenotomy that was successfully treated with surgical excision.

## Case presentation

**Figure 1:**
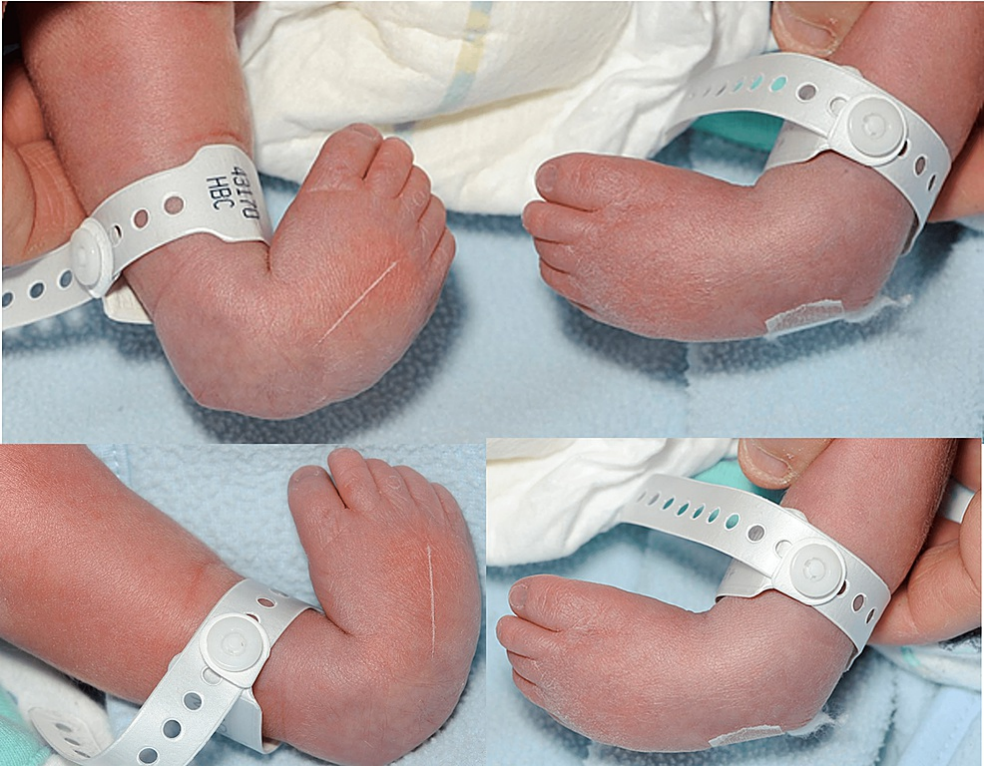
Clinical pictures showing talipes equinovarus at birth

**Figure 2:**
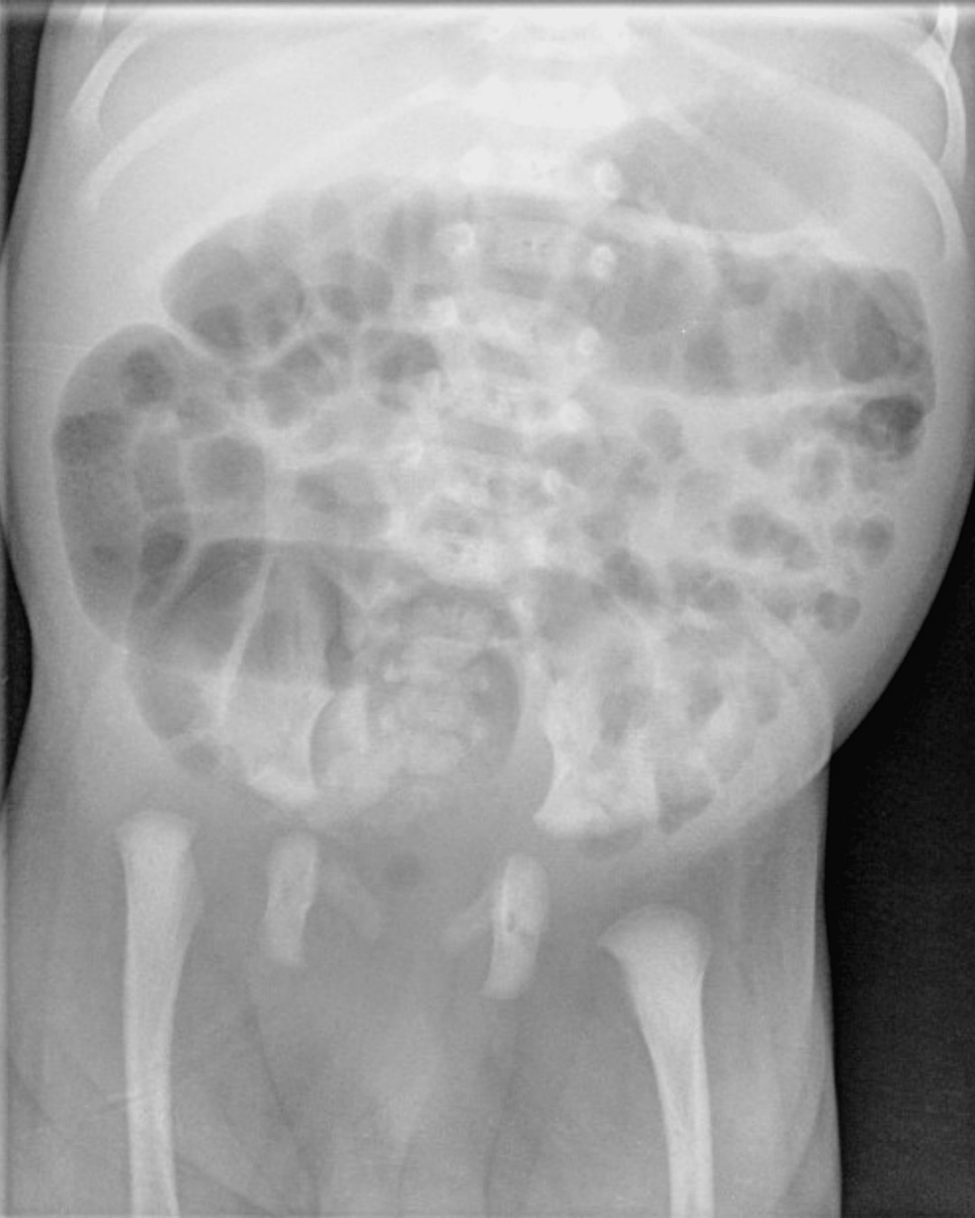
Pelvic radiograph at birth showing right hip joint dislocation

**Figure 3:**
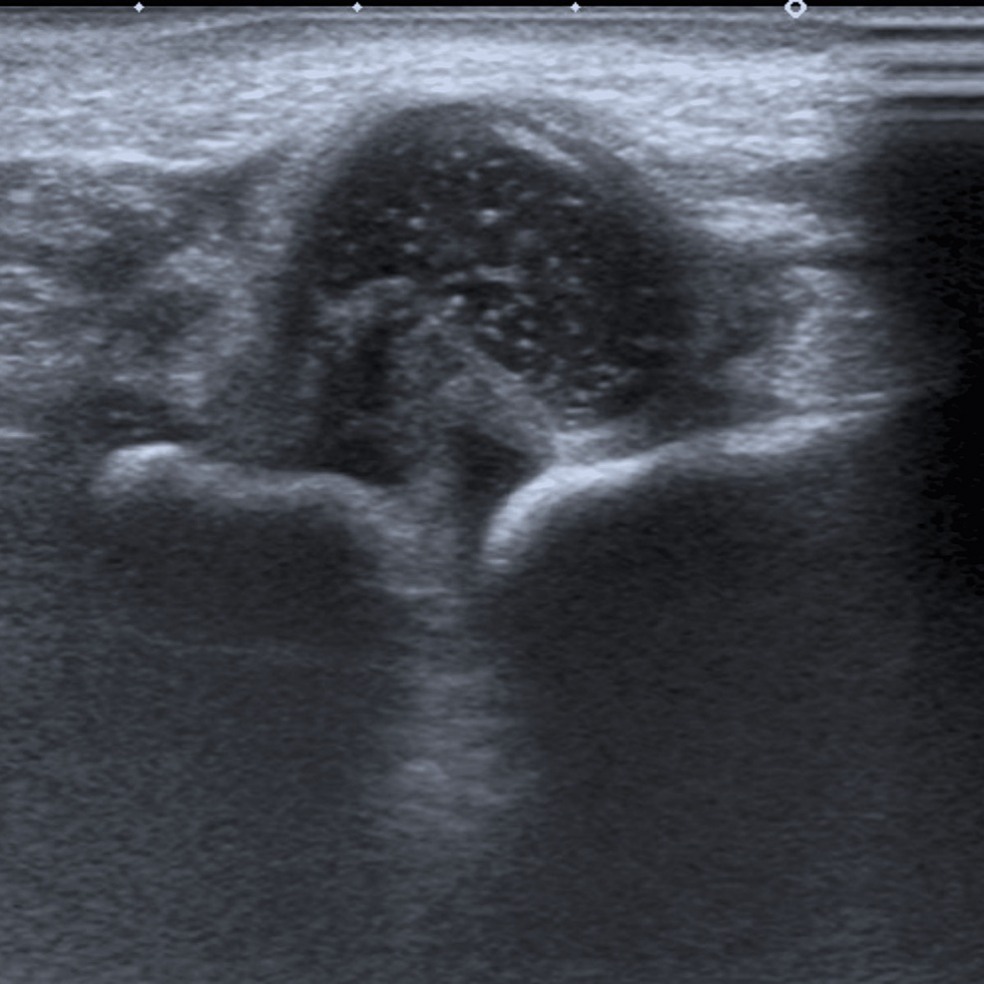
Ultrasound right hip at birth showing right hip dislocation

An 11-day-old baby born at caesarean section at 37 weeks was first seen in the paediatric orthopaedic clinic with bilateral talipes equinovarus, Pirani score of six bilaterally. There was no family history of clubfoot. At birth, pelvic and hip radiographs were performed as the paediatric team clinically identified limited abduction in his right hip during postnatal assessment, which demonstrated a dislocated right hip joint. Figure 1 and Figure 2 below show the patient's talipes and pelvic radiograph at birth. An ultrasound scan confirmed this, as seen in Figure 3. 

**Figure 4:**
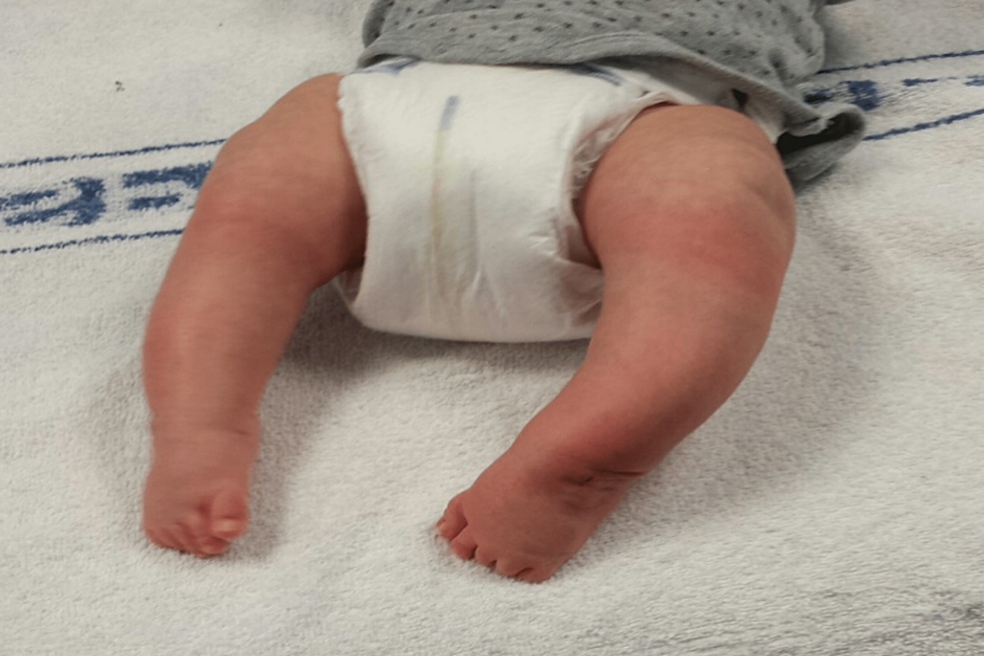
Pre-tenotomy, age 14 weeks

**Figure 5:**
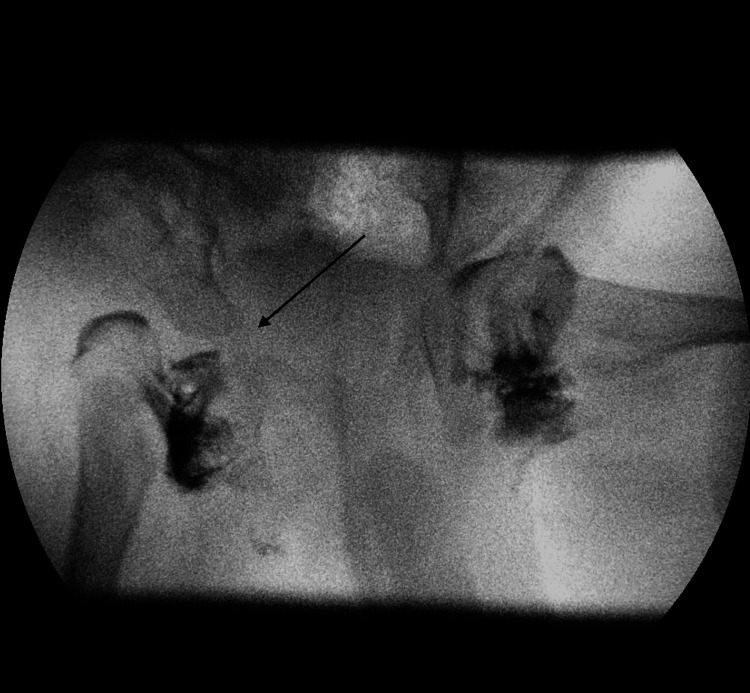
Hip arthrogram showing right hip joint dislocation

On examination he had global reduction in hip range of movement (ROM), raising the possibility of teratologic dislocation. He was treated with the Ponseti manipulative method for 12 weeks. Unfortunately his feet remained stiff, as shown in Figure 4, so he had bilateral tenotomies and hip arthrogram (Figure 5) at the same time to reduce his dislocated hip at three months.

Four hours after surgery he developed swelling in his toes and hence plaster had to be changed. He continued in his plaster and was reviewed weekly by the Ponseti physiotherapist making good progress.

**Figure 6:**
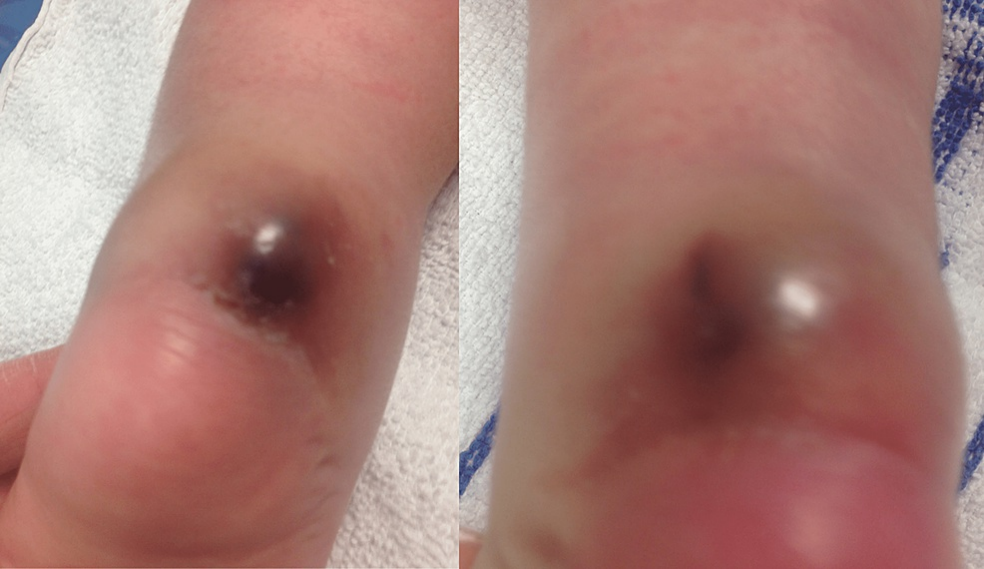
Two weeks post-tenotomy

**Figure 7:**
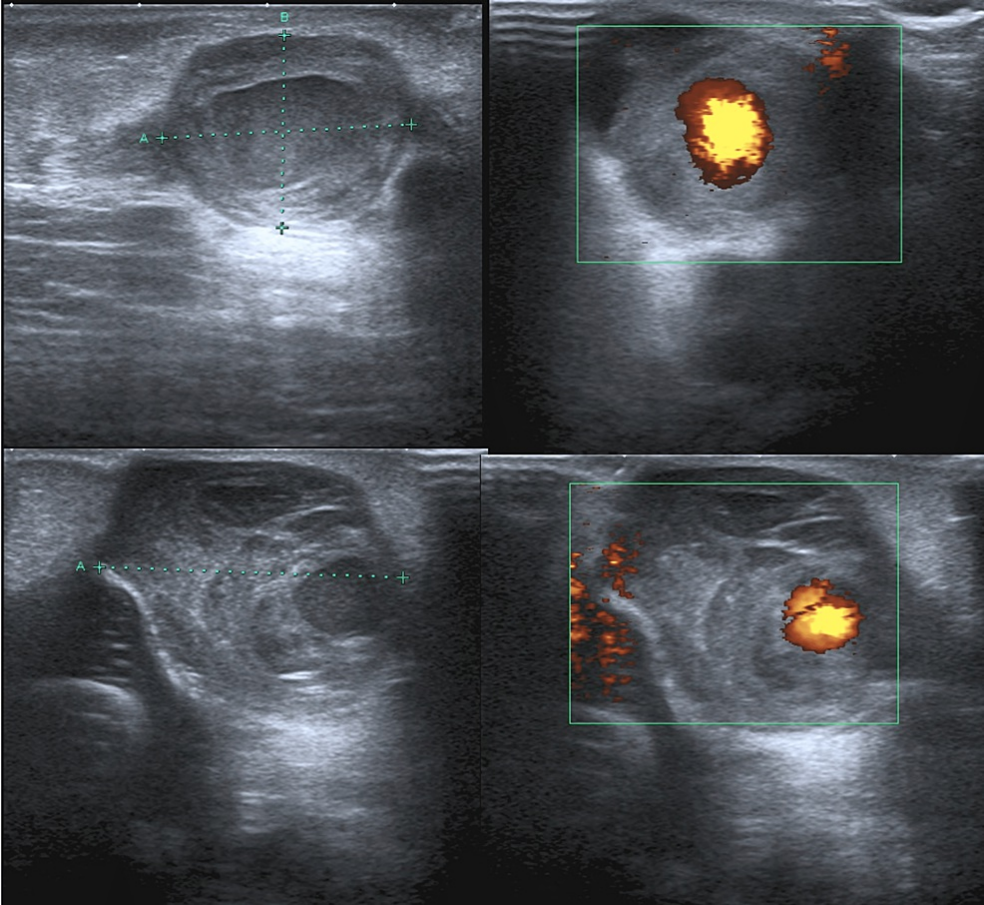
Colour Doppler ultrasound scan showing bilateral pseudoaneurysms

At week two post-tenotomies, swelling with bluish discolouration at the posterior aspect of both heels was noted (Figure 6). A colour Doppler ultrasound scan of the Achilles tendons on both sides confirmed almost identical bilateral posterior haematomas with pseudoaneurysm arising from the distal peroneal arteries (Figure 7). After discussing with colleagues, the treating surgeon decided to keep the feet out of cast for a week to allow for soft tissue healing before resuming below knee plaster cast bilaterally with pressure moulding focusing on the pseudoaneurysms. However at week four it was noted that the posterior swelling in his left foot had worsened and was necrotic looking above the tenotomy site. Further doppler ultrasound confirmed patent dorsalis pedis arteries on both sides.

**Figure 8:**
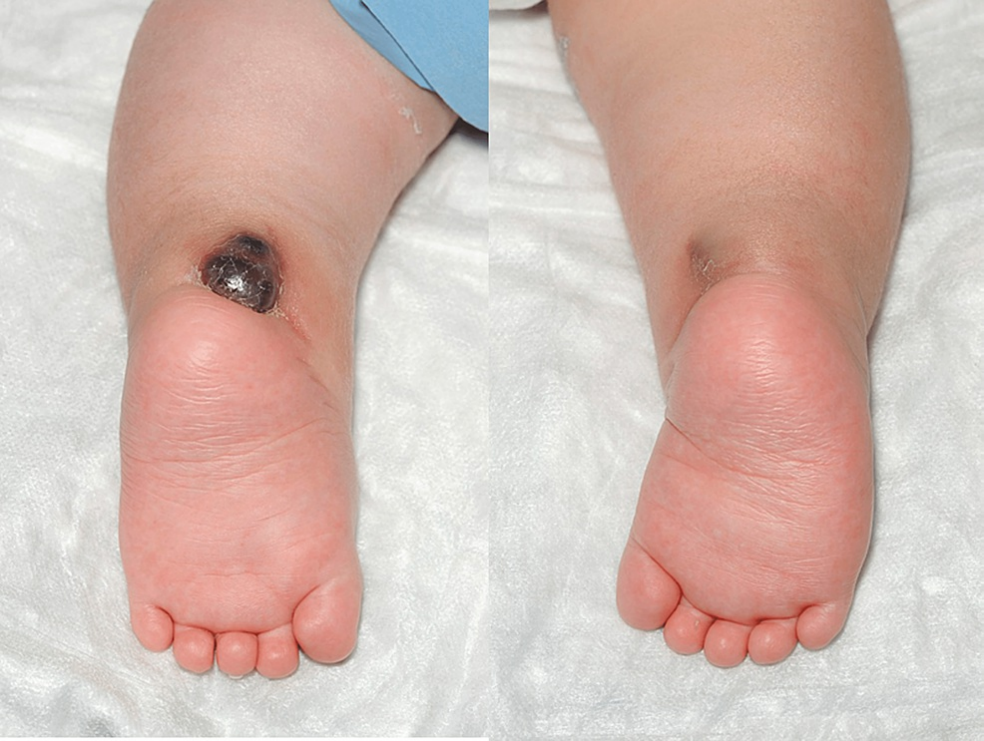
Bluish discolouration at week six post-tenotomy

**Figure 9:**
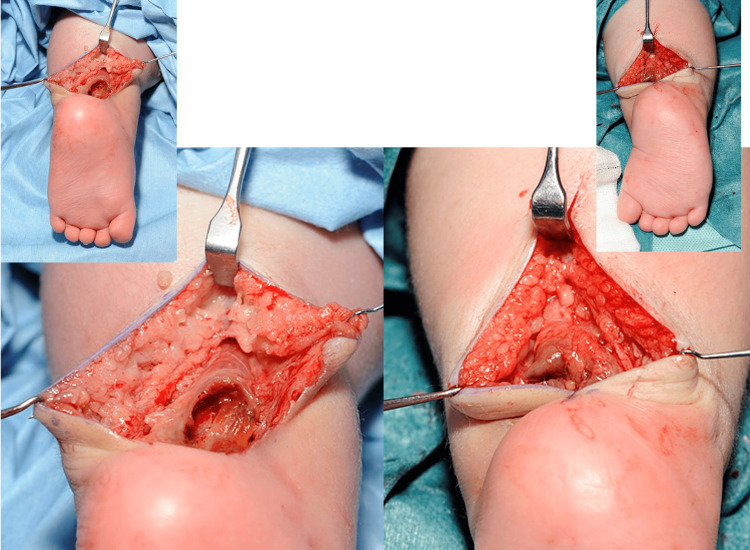
Pseudoaneurysm excision and haematoma evacuation

At week six as there was no clinical improvement in the swelling and discolouration with serial moulded casting (Figure 8), the bilateral pseudoaneurysms were surgically explored. Intra-operatively, bilateral peroneal arteries were found to be thrombosed with no active bleeding. Both pseudoaneurysms were excised, haematoma evacuated, and peroneal arteries ligated, followed by further Achilles tendon lengthening (Figure 9).

Two weeks post-exploration, although his left foot had a more limited range of movement, he was achieving 5° dorsiflexion and 20° abduction in both feet in above-knee plaster casts. At week three post-exploration, his right foot achieved 15-20° dorsiflexion and 20-30° abduction while the left foot achieved a plantigrade position with 20° abduction. His plaster casts were changed to a bar and boots orthosis (Mitchell-Ponseti foot abduction orthosis). 

## Discussion

**Figure 10:**
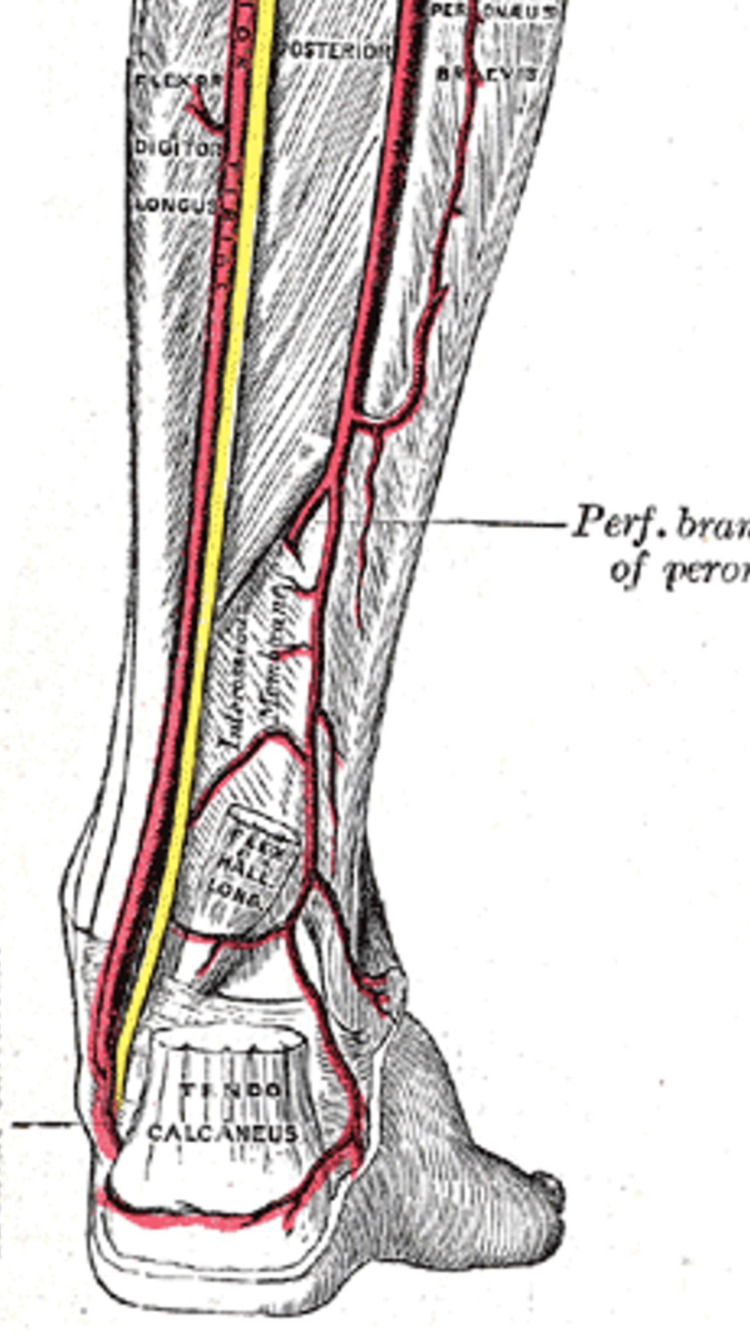
Posterior neurovascular anatomy at level of ankle

It is imperative to understand the anatomy involved when performing percutaneous tendoachilles tenotomy to prevent neurovascular injury. As shown in Figure 10, anterolateral to the Achilles tendon lies the peroneal artery, sural nerve, and lesser saphenous vein whereas anteromedially lies the posterior tibial neurovascular bundle [5]. Vascular anomalies in clubfoot have been recognised in 36% - 100% of cases [6,7] and have been proposed as an aetiology for congenital idiopathic clubfoot. The absence of anterior tibial artery [8,9] and posterior tibial artery [10-12] in children with severe clubfoot deformity have been reported. 

Pseudoaneurysm is a common vascular abnormality resulting from disruption in arterial wall continuity leading to a perfused sac contained by media, adventitia or soft tissue surrounding an injured vessel that communicates with the arterial system. This can be secondary to infection, trauma, or iatrogenic from surgical procedures [13]. In orthopaedics pseudoaneurysms have been reported following trauma such as an ankle sprain [14] and an ankle fracture [15], and following elective surgery such as ankle arthroscopy [16], release of plantar fascia, open clubfoot release [17], percutaneous multiple-cut tendon lengthening [18], and insertion of arterial cannula [19]. 

Dobbs et al. in 2004 retrospectively reviewed 219 idiopathic CTEVs of 134 infants of which 200 CTEVs required percutaneous tendoachilles tenotomy. They were the first to report four cases with serious bleeding complications, three from presumed injury to the peroneal arteries and one from injury to the lesser saphenous vein [2]. In 2007, Changulani et al. reported a case of laceration to the posterior tibial artery and complete transection of tibial nerve following a percutaneous tendoachilles tenotomy that was treated with ligation of the vessel and primary repair of the nerve [20]. 

The first case of pseudoaneurysm after Ponseti percutaneous Achilles tenotomy was reported in 2008 by Burghardt et al. [21]. They described a swelling that was noted behind the left Achilles tendon after three weeks of casting post-tenotomy, which led to technical difficulty of bracing the feet in a foot abduction orthosis. On second opinion one month later, a pseudoaneurysm was diagnosed with colour Doppler ultrasonography, most likely due to partial laceration of an artery. The pseudoaneurysm had completely resolved after four weeks of serial casting, negating the need for invasive intervention. In our case, surgical exploration was performed after three weeks of pressure moulding due to ongoing discolouration of the swelling above the tenotomy site. Clinically this indicated a lack of resolution of both pseudoaneurysms, hence they were ligated and excised, and the patient recovered well.

Lewis et al. in 2013 described a post-operative pseudoaneurysm following Ponseti percutaneous Achilles tenotomy [22]. An eight-week-old boy presented with active bleeding from a ruptured pseudoaneurysm of the posterior tibial artery leading to haemorrhagic shock four weeks after tenotomy. After stabilisation, the posterior tibial artery was explored and ligated proximal and distal to the pseudoaneurysm, and the patient made a good recovery. In their case, a swelling had been noticed three weeks post-tenotomy, similar to the case from Burghardt et al., and initial treatment plan was non-operative management. 

Furthermore Rademan in 2021 reported a case study of a patient who suffered a pseudoaneurysm after tendoachilles tenotomy [23]. In that case too, a swelling was noted at three weeks follow-up in the area of incision. Following a multi-disciplinary discussion, the treating surgeon decided to manage the pseudoaneurysm non-operatively. The pseudoaneurysm ruptured on cast change at third follow-up in clinic, and the patient underwent emergency exploration and successful ligation of the bleeding peroneal artery. The author theorised that the survival of the patient and successful ligation of the ruptured vessel could have been largely affected by it occurring whilst the patient was in the outpatient department in hospital, where immediate resuscitation was available. 

To our knowledge, this is the first case to report complications of simultaneous bilateral pseudoaneurysms following Ponseti tendoachilles tenotomy on both sides. The tenotomies were carried out by the same operating surgeon, at one sitting, with no change in technique. As previously mentioned, pseudoaneurysms are a rare complication, and in view of a case that occurred on both sides we hypothesize that the pseudoaneurysms occurred due to underlying aberrant vascular anatomy in a child with clubfoot as compared to an intra-operative technical error. We conjecture the possibility of an aberrant peroneal artery perforating branch that crosses in close proximity to the Achilles tendon, which may be prone to injury at the time of tenotomy. Sora et al. in 2008 carried out an anatomic imaging study of the adult ankle, and reported the tibial nerve to be 11.8 ± 2.4 mm and the posterior tibial artery to be 16.7 ± 3.8 mm in depth from the Achilles tendon at the tibiotalar joint [24]. This further highlights the proximity of the neurovascular structures, especially in an infant or child. 

## Conclusions

Pseudoaneurysm following Ponseti tendoachilles tenotomy is a rare complication of an otherwise safe and routine procedure. Published case reports place timing of the clinical findings; swelling and discolouration secondary to potential pseudoaneurysm at around three weeks post-tenotomy, therefore a high index of suspicion needs to be maintained especially around this time period post-tenotomy, when the infant is reviewed after removal of casts. In a case of bilateral pseudoaneurysms in the same setting, we raise the possibility of an aberrant perforating branch of peroneal artery that may be prone to injury at time of tenotomy. Operative versus non-operative management of pseudoaneurysms in CTEV is still a debated issue and every case needs to be managed individually as these cases are rare, and can present an ongoing obstacle to successful ongoing Ponseti treatment and fitting of boots and bar orthoses.
